# Toothless Twentieth Centenarians, or Vegetarianism—Which?

**Published:** 1899-03

**Authors:** H. T. Harvey

**Affiliations:** Battle Creek, Mich.


### Toothless Twentieth Centenarians, or Vegetarianism—Which?

BY H. T. HARVEY, D.D.S., BATTLE CREEK, MICH.

Read before the Toledo Dental Society, January 13, 1899.

*Why are there forty thousand dentists in the world to-day, when, fifty years ago, there were perhaps not over one thousand?* Why this tremendous increase of nearly one thousand per year? We are informed that, prior to this century our medical brethren, together with barbers, were able to attend to all the dental disaffections of the people. That we are living in the most significant era of modern civilization and setting the most rapid pace of all other races, I do not think you will deny. That disease in its every aspect is making just as rapid strides, I do not think can be contradicted. That there is a marked increase in the decay and consequent loss of the teeth in civilized countries, resulting from disease, either general or local in character, there can be no doubt.

Are we, as a race, going down physically, mentally and morally? In support of this proposition, I will give a few uncontrovertable facts.

There are at the present time, in the United States, nearly three times as many lunatics, three times as many imbeciles, twice as many drunkards, three times as many epileptics and twice as many criminals per thousand inhabitants as there were fifty years ago.

Please note that this increase is not simply numerical, but one of proportion. With the increase of population there would naturally come an increase of insane and other defectives, if there were not a gain in the physical stamina of the race. If the improved knowledge in sanitation and various advantages in the conditions of life which civilized human beings at this time enjoy were really having an effect to improve the vitality of the race, we ought to find the ratio of defectives, criminals, etc., as compared with sound and well-controlled individuals, diminishing rather than increasing.

An increase of 10 percent in the proportion of defectives, occurring within fifty years, would be a startling fact. It would mean, if continued long enough, complete race demoralization and extinction, but the increase of the past fifty years is an increase of nearly 300 percent, which is unmistakable evidence that the human race, even in the most civilized lands, and where are afforded the greatest freedom and the best conditions for physical, mental and moral health which any country offers (the United States) is going downward at a most appallingly rapid rate, and this deterioration is not simply physical, but moral and mental as well.

Disease has become more virulent than ever before. The great number of men, women and children confined in the counting-room, stores, factories and various sedentary occupations is developing a deformed creature, which may well be termed "the sedentary man" who is known by his round shoulders, his flat, hollow chest, weak heart, displaced stomach, puny muscles and his lusterless eye.

In former times cholera, the black plague and other dreaded cyclones of disease acted as a stay to degeneracy. These diseases spent their force upon the weak. Now we are protected by a sanitary cordon. Treacherous diseases knock at our doors in vain.

We cannot quarantine against tuberculosis, indigestion, heart failure and the many woes of our own finding.

What are the causes of these conditions? I do not wish to be known as an extremist, and will confine myself to reasonable causes as they appear to me.

From personal observation, the great cause of disease is excesses. That the use of intoxicating liquors, tobacco and the increase of sexual vices are great constitutional deteriorating causes there is no reason to doubt, but that through the depraved, gormandizing appetite of man eminating from his debased lower animal nature, his evil habits of life have their origin I am fully convinced. That constitutional deterioration, the result of improper nourishment resulting in systemic disease thereby lowering the vitality of the body, allowing germs of disease and decay to force into submission the strong and healthy organs, together with the teeth, resulting in their destruction is evident.

Decay of the teeth *does not* take place until this vital resistance is diminished to such a degree that they cannot combat the destructive influences of the germs of disease that come in contact with them.

Flesh, together with other indigestible foods, such as condiments, pastry, woody vegetable substances, etc., are, with the habits of *excesses*, the great cause of indigestion, which nine-tenths of the American people are afflicted with to-day. Indigestion is one of the greatest causes of constitutional deterioration, resulting in the decay and loss of the teeth.

Flesh is the most easily decomposed of all food materials, as you well know, both in the alimentary canal and in the mouth, and from which the greatest production of bacteria is obtained, resulting in fermentation-producing germs which destroy the structure of a tooth.

Dr. Brophy, while discussing a paper I read at Elkhart, Ind., last September, touching upon this subject, said: "Man was intended to eat meat," and to prove that we are of the carnivorous species, referred to the cuspid teeth of man as a characteristic of flesh-eating animals.

That man is not carnivorous by nature is proved by the very shape of the external frame of his body. He has no curved beak; no sharp talons and claws; no pointed teeth; no intense power of stomach or heat of blood which might help him to masticate and digest the gross and tough flesh substance. On the contrary, by the smoothness of his teeth, the small capacity of his mouth, the softness of his tongue and the sluggishness of his digestive apparatus, nature sternly forbids him to feed on flesh.

Man's mouth and teeth are a mill, formed upon the gradual reduction plan. Our front teeth furnish a perfect apparatus for cutting our food. Our side and back teeth are for grinding and pulverizing food. Flesh foods require very little mastication, therefore dogs, lions, etc., have teeth especially adapted for hetcheling and tearing meat. There can be no doubt that the use of flesh foods is one of the chief causes of dental decay. Little particles of meat get between the teeth and encourage the growth of destructive germs. The germs that destroy teeth are the same as those that cause the decay of flesh. It is better not to have dead things of any kind lying about in the mouth for the subsistance of germs. Keep it entirely free and clean from fragments of dead animals, and that can be most easily accomplished by putting nothing of the sort into the mouth.

The monkey is a vegetarian and has sound teeth, and I commend those who wish healthy and sound teeth to live on the monkey's diet—fruits, grains and nuts.

To convince you that flesh food is disadvantageous from the standpoint of nutrition, I will quote you a few facts: The total nutritive value of lean beef in 28 percent, containing 19.3 albuminous substance, 3.6 carbon, 5.1 salts; mutton contains 28 percent nutritive value; poultry, 26 percent; white fish, 22 percent; salmon, 23 percent; veal, 37 percent, and pork has 67 percent, but requires, in the roast, five hours and fifteen minutes to digest. The average total nutritive value of the seven varieties of flesh foods referred to is 33 percent, and the average time for the digestion of the same is four hours, forty-two minutes.

No wonder when one leaves the table after a hearty meal of Christmas fowl, (and it is all "foul") that you feel that you have had a square meal. The food has not had time to digest, nor the faithful stomach an opportunity to rest until the dose is repeated, and, ultimately, we find our stomach broken down, and we then wonder how it happened.

Why not take a meal of fruits and nuts, or of grains and nourishing vegetables, in which there is nearly three times the nutritive value and which reqnires less than half the time to digest, with the supreme knowledge that you are not supplying subsistence to numerous germ colonies, which will ruin your health and destroy your teeth?

Rice has a total nutritive value of 87 percent and requires only one hour to digest. Fruits, such as grapes, apples, pears, plums, cherries, etc., average about 15 percent in nutritive value and require about one hour and a half in which to digest, at the same time proving a perfect germicide for the teeth, mouth and alimentary canal. Nuts and grains are rich in nutritive elements and fats, both averaging 75 percent, and are easily digested.

Bad combinations of foods is another existing evil, which proves nearly as detrimental as the indigestible mass of flesh foods above referred to.

Vegetarianism is conducive to both health and longevity. Health is normal, health is absolute; there is but one nature. "The wisest care and fullest upbuilding of the body and its powers is a sacred duty, not lightly to be disregarded."

It is a very rare thing to find a dog with decayed teeth. Wolves, wild horses and other creatures that live close to nature have *good teeth.* Savages and primitive people, whose habits are simple, have good health and good teeth (except where they have come in contact with civilization, when they, too, have become contaminated), and do not require the services of a dentist.

The examples of great longevity and good health are all to be found in the lowly walks of life. Among peasants and common laborers, and a study of the habits of centenarians has shown them to be, without exception, persons of simple habits, and a majority of whom were abstainers from stimulating foods and drinks of all kinds.

Our prize fighters, long distance bicyclists, swimmers, long distance walkers cannot use meats and stimulants and win their contests, and records show that vegetarians always win contests where vital endurance plays any part.

I was interested in reading one day this week of a prima donna in Detroit who has been a strong vegetarian for the past eight years, through compulsion, and attributes the saving of her life to her reform in diet.

The world is waking up to the fact that something is radically wrong. The medical profession is casting side glances at the dietary problem. The people must have relief. The fetters of false ways, evil habits and depraved tastes are tightening about them and producing a demoralizing condition in society.

Where is the remedy? Begin at the foundation, build up new healthy tissue, with pure blood coursing through your veins by using wholesome, nourishing foods, and by correct habits of life. The race will then improve mentally and morally, and not until then.

Are there prospects of our race becoming toothless? I think there are, and our only salvation is the adoption of a vegetarian diet and exercise, through which medium the principles of hygiene and sanitation can be promulgated and adopted by the victims of error.

